# Adenovirus-mediated suppression of hypothalamic glucokinase affects feeding behavior

**DOI:** 10.1038/s41598-017-03928-x

**Published:** 2017-06-16

**Authors:** Romina María Uranga, Carola Millán, María José Barahona, Antonia Recabal, Magdiel Salgado, Fernando Martinez, Patricio Ordenes, Roberto Elizondo-Vega, Fernando Sepúlveda, Elena Uribe, María de los Ángeles García-Robles

**Affiliations:** 1 Departamento de Biología Celular, Universidad de Concepción, Concepción, Chile; 2 Departamento de Bioquímica y Biología Molecular, Facultad de Ciencias Biológicas, Universidad de Concepción, Concepción, Chile; 3 Instituto de Investigaciones Bioquímicas de Bahía Blanca, Universidad Nacional del Sur, y Consejo Nacional de Investigaciones Científicas y Técnicas, Bahía Blanca, Argentina; 4 Facultad de Artes Liberales, Facultad de Ingeniería y Ciencias, Universidad Adolfo Ibáñez, Viña del Mar Chile, Chile

**Keywords:** Immunological techniques, Cellular neuroscience

## Abstract

Glucokinase (GK), the hexokinase involved in glucosensing in pancreatic β-cells, is also expressed in arcuate nucleus (AN) neurons and hypothalamic tanycytes, the cells that surround the basal third ventricle (3V). Several lines of evidence suggest that tanycytes may be involved in the regulation of energy homeostasis. Tanycytes have extended cell processes that contact the feeding-regulating neurons in the AN, particularly, agouti-related protein (AgRP), neuropeptide Y (NPY), cocaine- and amphetamine-regulated transcript (CART) and proopiomelanocortin (POMC) neurons. In this study, we developed an adenovirus expressing GK shRNA to inhibit GK expression *in vivo*. When injected into the 3V of rats, this adenovirus preferentially transduced tanycytes. qRT-PCR and Western blot assays confirmed GK mRNA and protein levels were lower in GK knockdown animals compared to the controls. In response to an intracerebroventricular glucose injection, the mRNA levels of anorexigenic POMC and CART and orexigenic AgRP and NPY neuropeptides were altered in GK knockdown animals. Similarly, food intake, meal duration, frequency of eating events and the cumulative eating time were increased, whereas the intervals between meals were decreased in GK knockdown rats, suggesting a decrease in satiety. Thus, GK expression in the ventricular cells appears to play an important role in feeding behavior.

## Introduction

As one of the main centers controlling food intake, the hypothalamus is a structure located in the basal region of the third ventricle (3V), and is composed of neurons (grouped in different nuclei) and other cells, such as tanycytes, that respond to glucose concentration^1–4^. Tanycytes are specialized ependymal cells that cover the lower lateral walls and the floor of the 3V. Morphologically, tanycytes are polarized, lengthened cells with apical surface differentiations in direct contact with the cerebrospinal fluid (CSF) and long basal processes that come into close contact with neurons from the periventricular nuclei of the basal hypothalamus^5^. Based on their morphological and histochemical features, tanycytes have been classified into four subpopulations: α1, α2, β1 and β2^6^. α2 and β1 tanycytes are localized in the lower lateral wall of the 3V. Importantly, they have extended cell processes that project into the arcuate nucleus (AN) and appear to come into close contact with feeding-regulating neurons, particularly agouti-related protein (AgRP), neuropeptide Y (NPY), cocaine- and amphetamine-regulated transcript (CART) and proopiomelanocortin (POMC) neurons^7, 8^. β2 tanycytes are located in the floor of the 3V. lining the median eminence (ME). These cells develop tight junctions that form the CSF-ME barrier. The proximal part of the cells is in contact with the CSF whereas the distal part of the cells forms processes in which the end feet reach the local capillary plexus in the ME. Although the specific roles of tanycytes in the hypothalamus are still under debate, their strategic proximity to and relationship with fenestrated capillaries, the blood brain barrier, axonal nerve terminals, and hypothalamic nuclei that regulate appetite/energy expenditure has placed them in a privileged position to integrate multiple inputs and regulate homeostasis^9^.

The pancreas, liver and small intestine are specialized organs, which respond to changes in blood glucose levels. The presence of glucose transporter 2 (GLUT2) and glucokinase (GK, hexokinase IV) enables glucosensing cells to detect blood glucose levels^10, 11^. We and others have previously demonstrated that tanycytes express several proteins involved in the peripheral glucosensing mechanisms, such as GK (the pancreatic isoform), GLUT2 and the pore-forming subunit of ATP-sensitive potassium channels, Kir6.1^12–14^.

In addition to expression, GK activity has been observed in tanycytes (S_0.5_ of 10 mM)^15^. Thus, it is the presence of this functional isoform of GK, together with GLUT2, that makes it possible for tanycytes to sense and respond to glucose changes with increased intracellular calcium in a mechanism that involves GK activity^3^. The fact that GK knock-out mice die a few days after birth due to acute hyperglycemia highlights the vital role of GK^16, 17^. Millán *et al*.^18^ reported hypothalamic GK to be expressed mainly in α and β tanycytes; GK expression was weak in neurons and absent in astrocytes^18^. Similarly, Salgado *et al*.^15^ cloned GK expressed in tanycytes and demonstrated its functionality. However, there is enough information that validates its location in hypothalamic neurons^19–22^. Additionally, GLUT2 is expressed in hypothalamic tanycytes^12^. Interestingly, hypothalamic AN neurons do not express GLUT2; however, they are connected to nerve endings from GLUT2-expressing neurons^23^. On the other hand, GLUT2 is so well-located in the nucleus of solitary tract in neurons, and there is no evidence of glia expression in this brain region^24^. Recent evidence obtained after molecular and pharmacological activation of GK in rats cannulated into the AN shows that GK activity specifically regulates glucose intake and dietary glucose uptake^25^. Expression of glucokinase regulatory protein (GKRP), a known GK inhibitor with a critical metabolic role in hepatocytes^26^, has also been demonstrated in tanycytes^15^. The presence of GKRP in tanycytes reinforces the importance of GK activity in these cells.

Over the last five decades, it has been suggested that glucose availability may regulate food intake. Despite the considerable efforts invested in unraveling the molecular mechanisms related to hypothalamic glucosensing and satiety response, there are still many unanswered questions. Here, we show that injection of an adenovirus expressing short hairpin RNA (shRNA) targeting GK into the 3V generates specific transduction of ependymal glia, especially tanycytes. GK knockdown animals showed no variation in NPY and AgRP (orexigenic neuropeptides) and CART (anorexigenic). POMC (another anorexigenic neuropeptide) showed to be even decreased in response to glucose when GK was knocked down. In conclusion, knockdown animals lost the normal response to glucose (i.e. a decrease in orexigenic neuropeptides and an increase in anorexigenic neuropeptides). In addition, alterations in feeding behavior (i.e., decreased satiety) were detected. Based on these results, we postulate that hypothalamic GK activity could regulate food intake as part of a glucostatic control system.

## Results

### Ad-shGK-EGFP injection into the basal 3V

**Figure 1:**
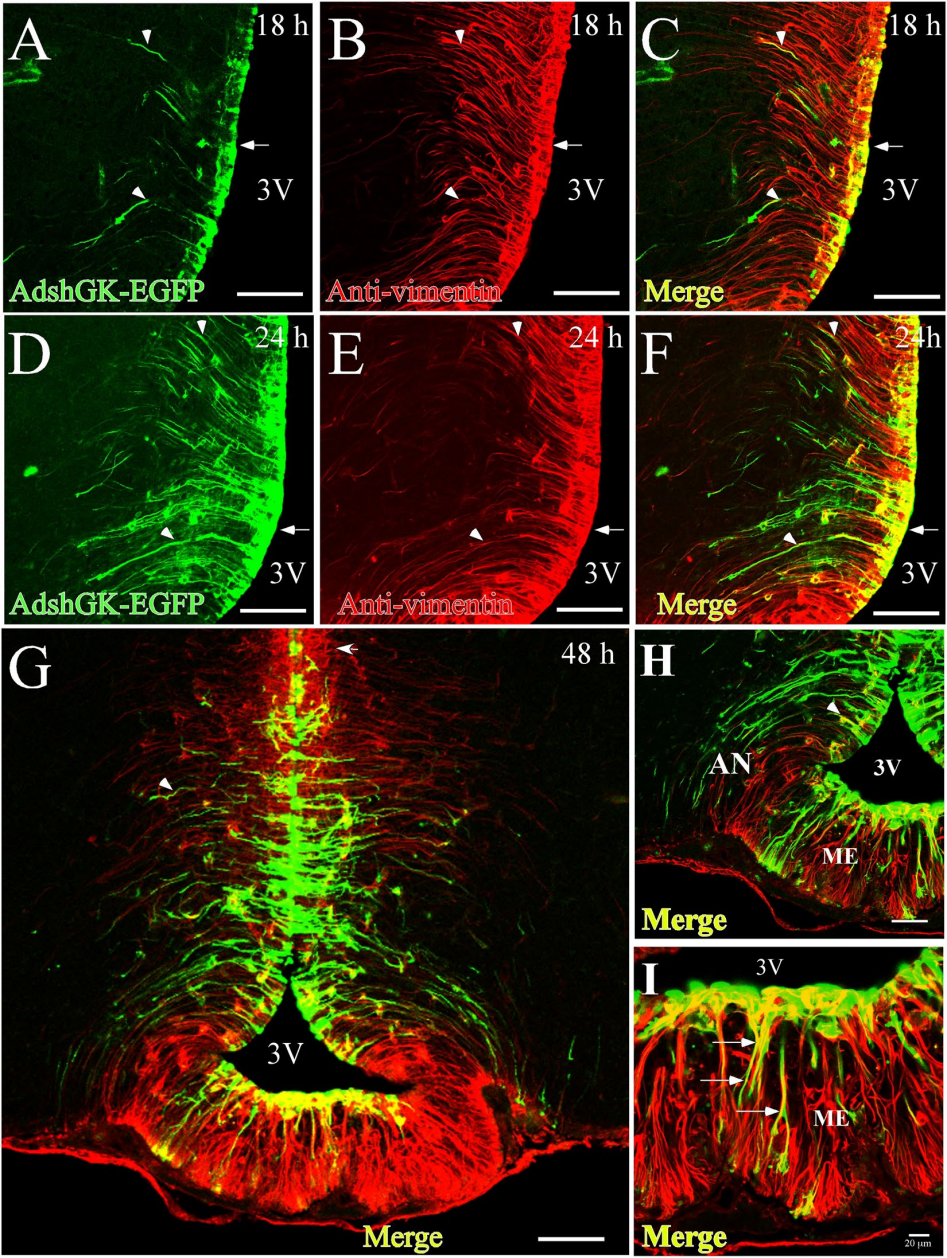
Time-course of adenoviral transduction in the basal hypothalamus. (**A–L**) Frontal sections (40 µm) from the hypothalamus at 18 h (**A–C**), 24 h (**D–F**) and 48 h (**G–I**) following injection of adenoviral particles (Ad-shGK-EGFP) into the 3V. EGFP fluorescence (green) and immunoreactivity for vimentin (red) and colocalization of EGFP and vimentin (yellow) are shown. Arrows show the processes of transduced tanycytes. (**G**) Panoramic view of the basal hypothalamus, showing tanycyte transduction. (**I**) Higher magnification image showing that Ad-EGFP transduction occurs in β2-tanycytes. AN, arcuate nucleus; 3V, third ventricle; ME, median eminence. Scale bars (**A**–**F** and **H**) 50 µm, (**G**) 100 µm, (**I**) 20 µm.

Since the success in GK knockdown depends upon the efficiency of adenoviral transduction, time-course studies were carried out in order to determine the time point at which there was a maximal infection rate. We analyzed EGFP fluorescence levels in rat brain slices at 18, 24 and 48 h post-i.c.v. injection of the recombinant adenovirus and compared them with levels of the tanycyte marker, vimentin, by immunostaining (Fig. 1). At 12 h post-injection, a faint signal from EGFP was detected in a few α-tanycytes (data not shown). At 18 h after i.c.v. injection, EGFP expression was detected in ventricular cells with elongated processes, which due to their location, correspond to α-tanycytes (Fig. 1A, arrow), which are vimentin-positive (Fig. 1B,C). High fluorescence was detected in the proximal region and basal processes of vimentin-positive cells at 24 h after injection (Fig. 1D–F). High levels of fluorescence were still observed for at least 48 h post-i.c.v. injection in ependymal cells (1), α-tanycytes, (2), β1-tanycytes (3) and β2-tanycytes (4) (Fig. 1G). Higher magnification images are shown in Fig. 1H and I. Importantly, EGFP fluorescence was limited to tanycytes, and no transduction of other areas was observed. EGFP was detected in the cell bodies and processes of cells lining the infundibular recess, which according to their location, correspond to α and β1-tanycytes. Particularly, the colocalization of Ad-EGFP with vimentin (shown in yellow) became more evident in β2-tanycytes (Fig. 1G–I).

**Figure 2:**
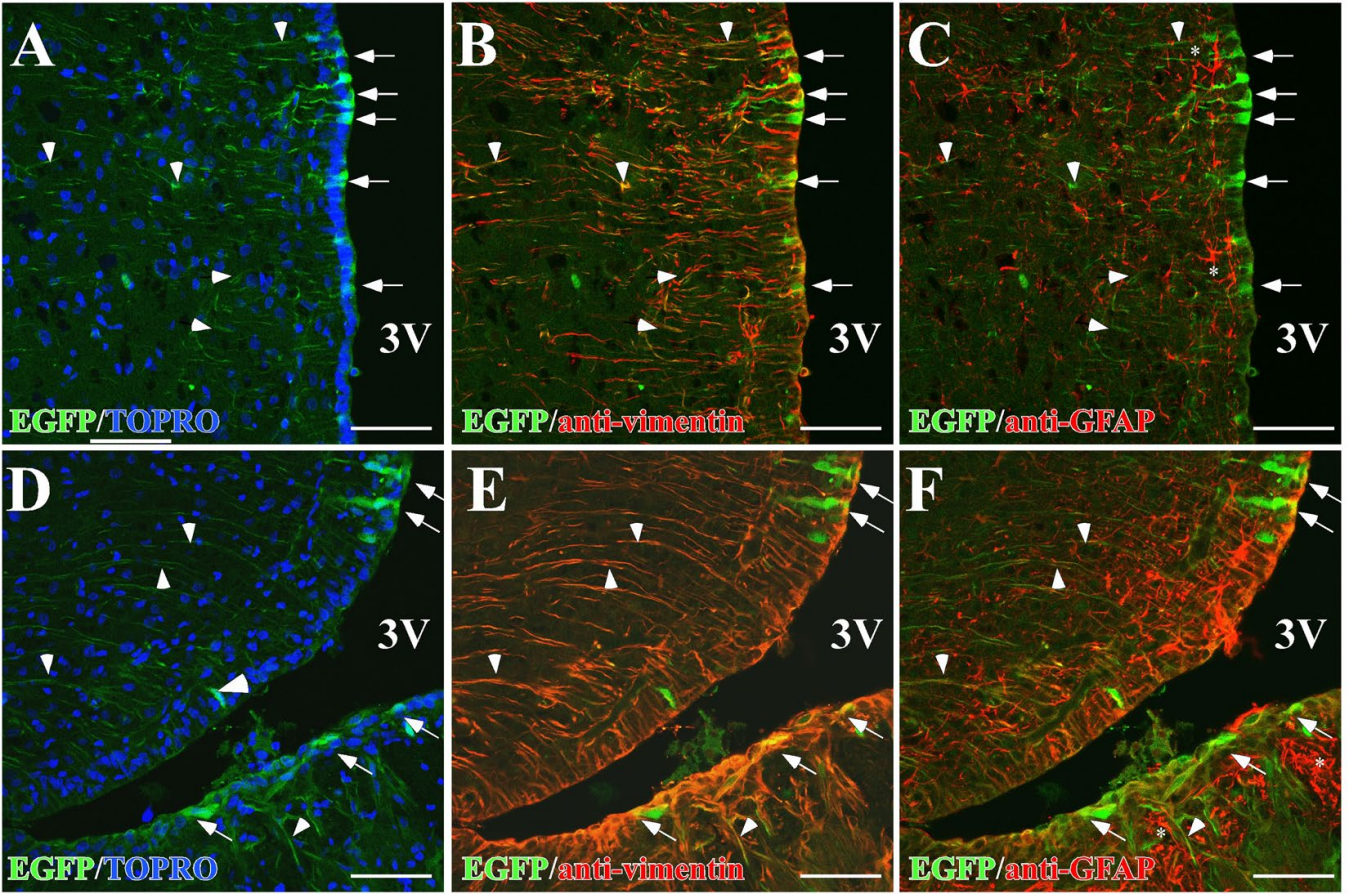
Distribution of EGFP in hypothalamic glial cells. (**A–F**) Representative confocal images of one focal plane using anti-vimentin (red) or anti-GFAP (red) antibodies as well as the fluorescence associated with EGFP (green) and the nuclear marker, TOPRO-3 (blue). (**A–C**) Adenoviral transduction in α-tanycytes. (**D–F**) Adenoviral transduction of β-tanycytes. (**A**,**C**) EGFP detected in apical membranes (arrows) and in long cellular processes in the basal hypothalamus (arrowheads). (**B**,**E**) Vimentin showing both proximal (arrows) and distal (arrowheads) tanycyte regions and colocalization with the EGFP. (**C**,**F**) GFAP showing astrocyte distribution, the absence of GFAP in the ventricular wall (arrows) and EGFP-positive processes (arrowheads). Scale bars (**A–H**) 50 µm.

**Figure 3:**
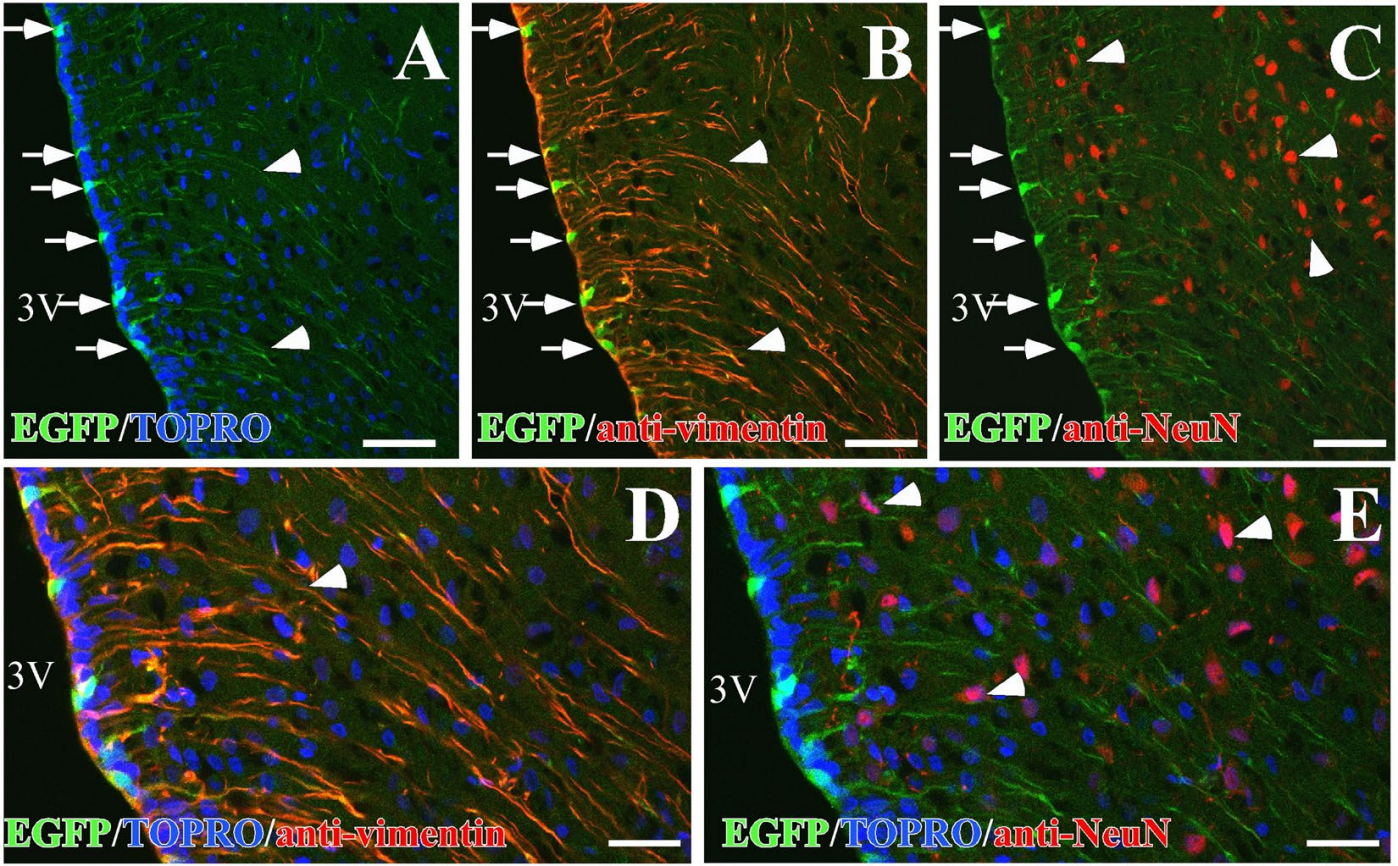
Co-distribution of EGFP and the neuronal marker, NeuN, in the basal hypothalamus. (**A–E**) Representative confocal images of one focal plane using anti-vimentin (red) or anti-NeuN (red) antibodies as well as EGFP fluorescence (green) and TOPRO-3 (blue). (**A**) Image showing EGFP in an α-/β-tanycyte transition region. (**B**) EGFP detected in the proximal (arrows) and distal processes (arrowheads) of vimentin-positive α-and β-tanycytes. (**C**) The neuronal marker, NeuN, distribution (red), showing the absence of colocalization with EGFP. (**D**,**E**) High magnification image of the area shown in (**B** and **C**). Scale bars (**A–C**) 50 μm; (**D**,**E**) 25 μm.

EGFP fluorescence was detected in cells whose localization and morphological features are suggestive of tanycytes. We evaluated astrocyte transduction in a three dimensional reconstruction of 20 focal planes (Supplementary Fig. S1). Sections of the basal hypothalamus from transduced animals were analyzed by spectral confocal microscopy for detecting EGFP fluorescence (green) and the astrocyte markers, GFAP (blue) and vimentin (red). EGFP expression was detected in the ventricular cells having long cellular processes, which due to their location, are α- and β-tanycytes. The expression of GFAP and vimentin was also detectable in the same region, where a different distribution of both proteins was clearly observed. In agreement with previous results^27^, GFAP was mainly detected in the subependymal region of the ME. Analysis of the co-expression of EGFP and GFAP at higher magnification revealed no co-expression of both proteins. However, a clear co-distribution of EGFP and vimentin was observed in the proximal region (arrowhead) and processes of tanycytes (arrows). Because adenoviruses can cause gliosis, which favors the incorporation of adenovirus by reactive astrocytes, we next evaluated the colocalization of both glial markers using a focal plane and Pearson’s correlation analysis. Colocalization of EGFP with vimentin was detected in the ventricular area (Fig. 2B,E, arrows) and in several cell processes (Fig. 2B,E, arrowheads). The Rr value estimated for EGFP/vimentin colocalization was 0.47 ± 0.11 in the area of α-tanycytes (Fig. 2B), whereas the Rr value for EGFP/GFAP colocalization in the same region was 0.18 ± 0.02, suggesting that EGFP colocalized to a larger extent with vimentin as compared with GFAP (Fig. 2C). Intense EGFP and vimentin colocalization (yellow) was observed in β-tanycytes (Fig. 2E, arrows) and the long fine processes contacting AN neurons (Fig. 2E, arrowheads). In this region, the Rr value estimated for EGFP/vimentin colocalization was 0.53 ± 0.06, whereas the Rr value estimated for EGFP/GFAP colocalization was 0.01 ± 0.02 (Fig. 2F). Astrocytes located in the proximity of α-tanycytes were strongly positive for GFAP, but not for EGFP (Fig. 2C, asterisks). The absence of transduction was detected also in astrocytes located in the proximity of β1-tanycytes and β2-tanycytes from the ME (Fig. 2F, asterisks). Although other authors have reported the serotype 5 adenoviruses transduces astrocytes, our results suggest that if adenovirus is injected in the 3V, it preferentially transduced tanycytes and not astrocytes. We used the neuronal marker, NeuN, and the tanycyte marker, vimentin, to evaluate neuronal transduction (Fig. 3A–E). EGFP was absent in NeuN-positive neurons (Fig. 3C,E), suggesting that AN neurons were not transduced with the adenovirus. Additionally, we analyzed the EGFP fluorescence in other cerebral regions, such as the cortex, hippocampus, and choroidal plexus, and compared it with that observed in the ventricular walls of the same animals. The intensity of EGFP fluorescence was systematically different in the tanycyte population with most fluorescence detected in β2-tanycytes then α-tanycytes and slightly lower in β1-tanycytes. Similar to β2-tanycytes, EGFP was detected in 3V-ependymal cells with weak signal observed in the choroid plexus and no signal detected in the cortex and hippocampus (Supplementary Fig. S2).

### *In vivo* GK inhibition by adenoviral injection into the 3V

**Figure 4:**
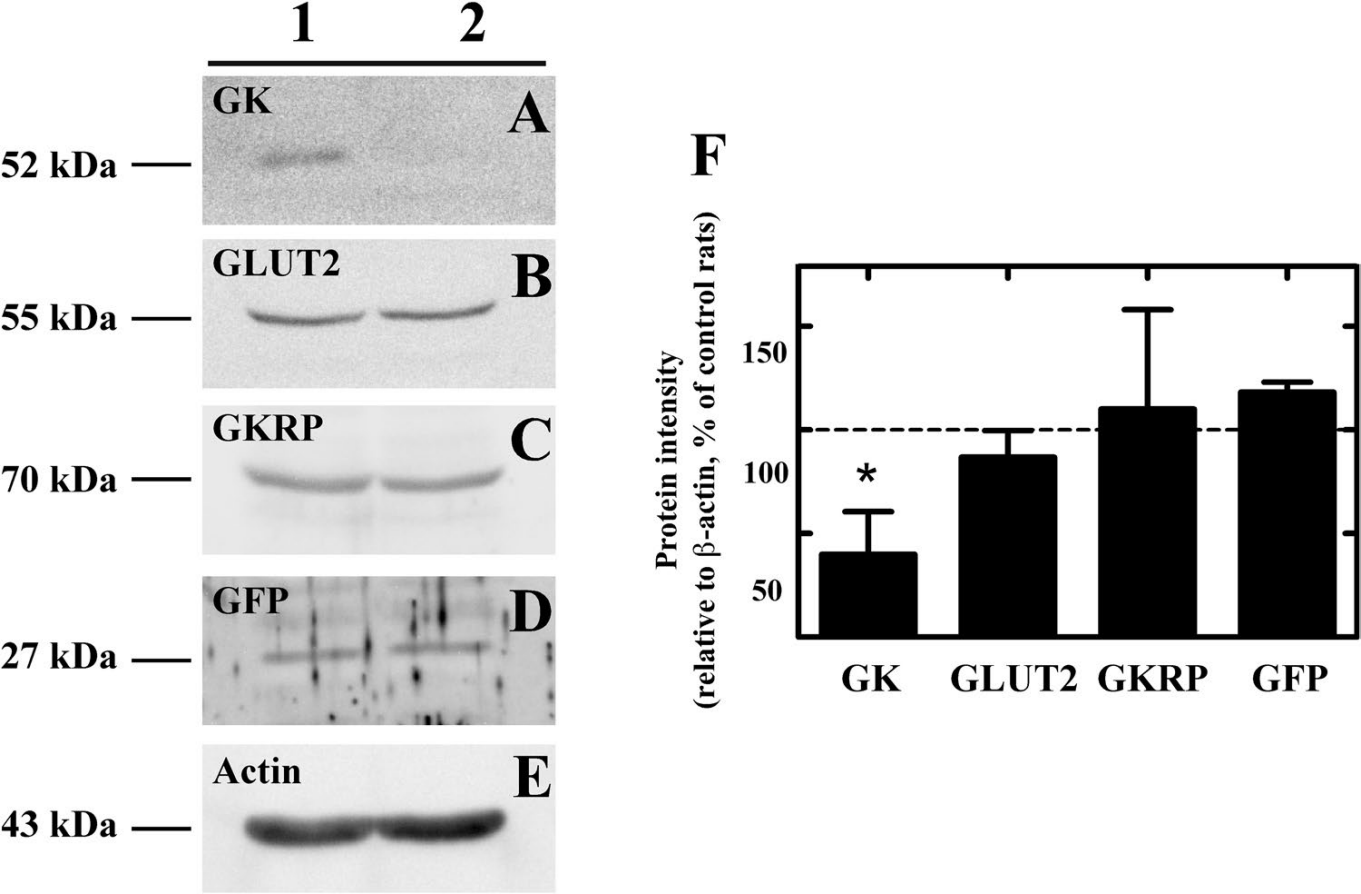
GK inhibition 48 h after AdshGK injection into the 3V. (**A**) Western blot assays of hypothalamic protein extracts in control (Ad-shβGal-injected rats) and Ad-shGK-injected rats using anti-GK, anti-GLUT2, anti-GKRP, and anti-GFP (transduction control) antibodies. An anti-β-actin antibody was used as loading control. Images are representative of three different experiments. (**B**) Densitometric analysis of each protein relative to β-actin evaluated in Ad-shGK-injected rats, as a percentage of this protein in Ad-shβgal-EGFP-injected rats. Statistics: t-test with Welch correction *p < 0.05.

We evaluated GK expression in total hypothalamic protein extracts at 48 h post-transduction using Western blot analysis of hypothalamic samples from rats injected with Ad-shβGal-EGFP or Ad-shGK-EGFP. As shown in Fig. 4A, GK expression was found to be significantly decreased (75%) in rats injected with AdshGK-EGFP with respect to Ad-shβGal-EGFP-injected rats. We also evaluated whether GLUT2 expression, which is also present in tanycytes^12^, was altered by GK inhibition. We detected no significant differences in protein levels between both animal groups, demonstrating the specificity of the GK shRNA (Fig. 4B). Similarly, GKRP, which is also expressed in tanycytes^15^, was not affected by GK inhibition (Fig. 4C). EGFP was detected at similar levels in rats transduced with both adenoviruses (Fig. 4D). The intensity of the bands was normalized to β-actin and expressed as a percentage of the ratio obtained with the control. Thus, only GK expression was significantly decreased following injection of the adenovirus carrying the shGK-RNA, and no compensatory effects on GLUT2 or GKRP expression occur under these conditions. Finally, immunohistochemistry analysis in the basal hypothalamus in both Ad- shGK and Ad-shβgal using anti-GK and anti-vimentin antibodies demonstrated a minor degree of GK fluorescence in GK knockdown animals. In these animals, GK-associated fluorescence was only decreased in vimentin-positive cells; no differences were detected in AN neurons (Supplementary Fig. S3).

### Effect of GK knockdown on neuropeptide expression in response to i.c.v. glucose injection

**Figure 5:**
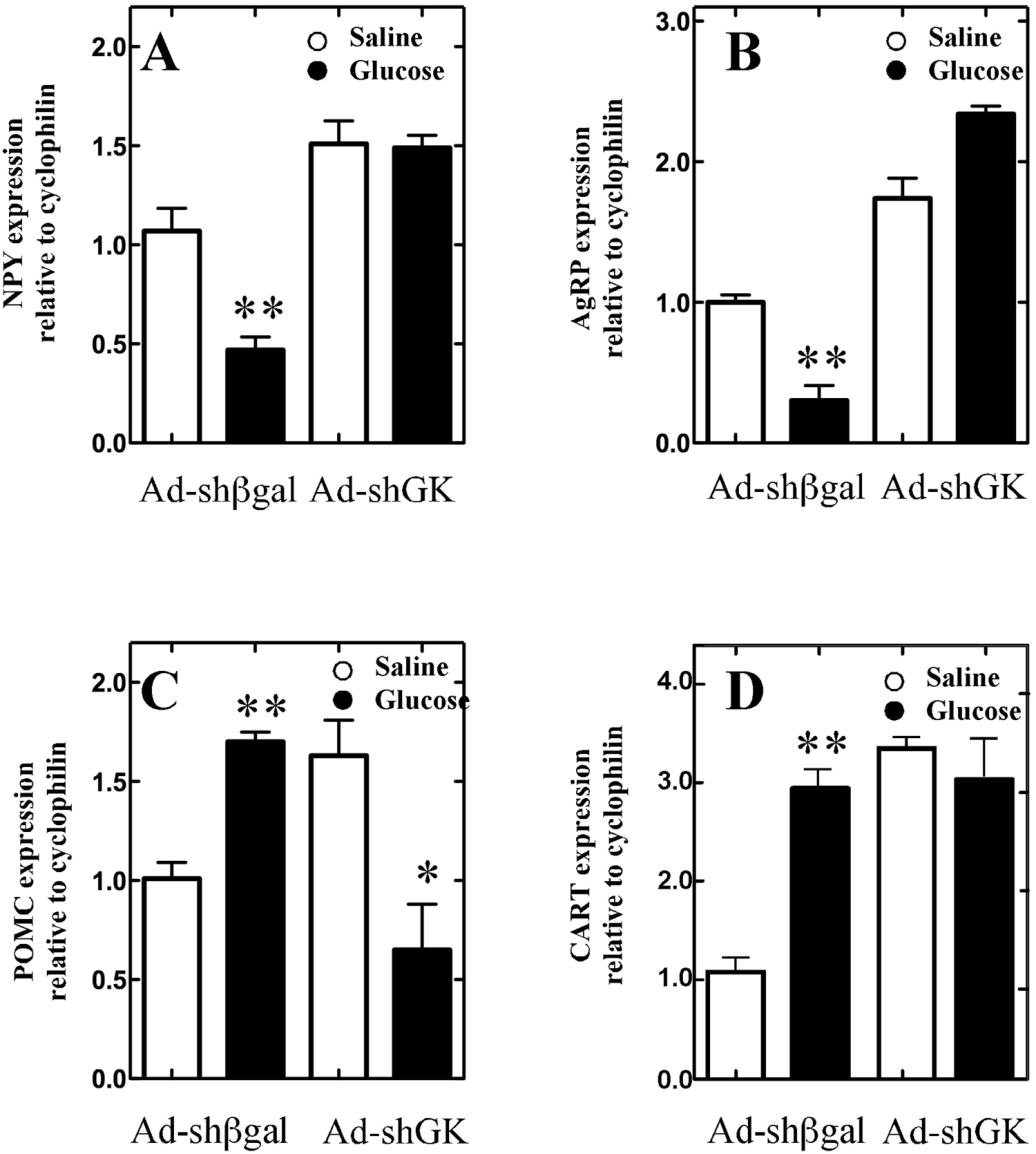
Expression of neuropeptides involved in feeding regulation. (**A–D**) Control (Ad-shβGal-EGFP-injected animals) and AdshGK-injected animals (Ad-shGK) were subjected to a 24-h fasting period. After that, saline or D-glucose was injected into the 3 V. After 2 h, NPY (**A**), AgRP (**B**), POMC (**C**) and CART (**D**) mRNA levels were assessed via qRT-PCR. Data are relative to cyclophilin (media ± SEM; n = 6) and processed by the ΔΔCT method. Statistics: Non-parametric ANOVA; *p < 0.05.

It has been previously shown that a 50 mM glucose stimulus directly into the 3V generates a neuronal response mediated by changes in the expression of anorexigenic and orexigenic neuropeptides^28^. Here, we tested if this glucose-triggered response was maintained in rats following GK knockdown. At 48 h after transduction, rats were subjected to a 24-h fasting period. Control and GK knockdown rats were next i.c.v. injected with 50 mM D-glucose (or saline). At 2 h after the glucose challenge, the expression of feeding-regulatory neuropeptides was analyzed by qRT-PCR (Fig. 5A–D). After glucose injection, control rats (Ad-shβGal-EGFP) showed a 50% and 70% decrease in the orexigenic peptides, NPY and AgRP, respectively (Fig. 5A,B, respectively) with respect to saline treatment. In contrast, a 70% and 200% increase in the anorexigenic peptides, POMC and CART, respectively, was observed in glucose-injected control rats compared to saline-injected control rats (Fig. 5C,D, respectively). This decrease in orexigenic neuropeptides and increase in anorexigenic neuropeptides is the expected normal response to glucose^29^. However, when saline and glucose treatment responses were compared in GK knockdown animals, no statistically significant differences were detected in orexigenic neuropeptides (Fig. 5A,B). Regarding anorexigenic neuropeptides, CART expression showed no difference between saline-injected and glucose-injected GK knockdown animals (Fig. 5D). However, POMC expression was decreased in glucose-injected GK knockdown animals compared to saline-injected GK knockdown animals (Fig. 5C). Saline-injected animals (control and GK-inhibited) showed significant differences in anorexigenic neuropeptides, which suggest that GK could be participating in the normal response to starvation. These results clearly indicate that GK knockdown animals lost the glucose-regulated expression of feeding-regulating neuropeptides.

### Effect of hypothalamic GK knockdown on food intake and feeding behavior

**Figure 6:**
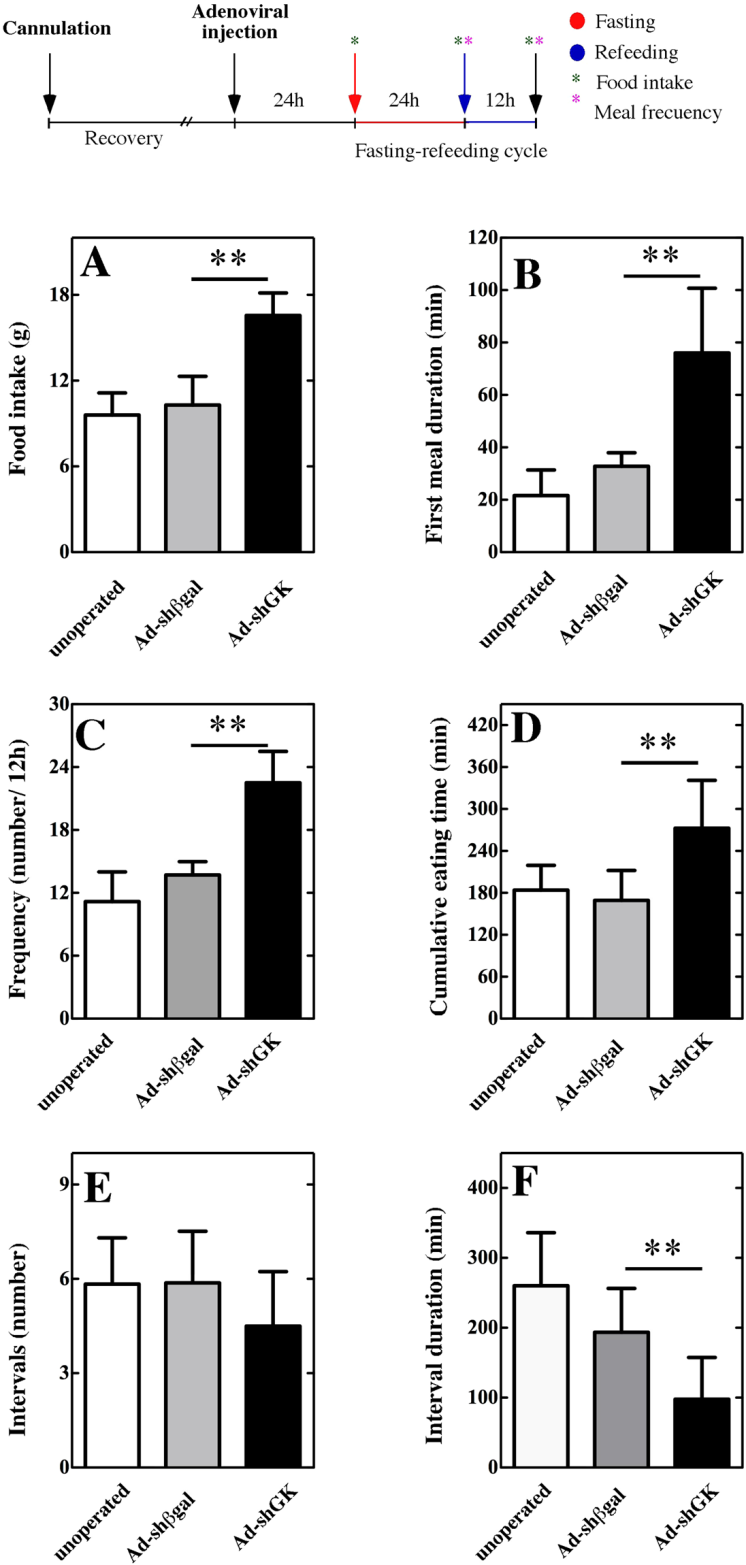
Effect of GK knockdown on feeding behavior in control and GK knockdown animals. (**A–F**) Food intake (**A**), first meal duration (**B**), frequency of eating events (**C**), cumulative eating time (**D**), intervals and interval duration (**F**) were evaluated in unoperated, Ad-EGFP-injected (Ad-EGFP) and Ad-shGK-injected animals (Ad-shGK). Schematic representation of the experimental procedure is shown in the top. Statistics: non-parametric ANOVA; **p < 0.01.

After confirming that the GK gene was preferently knocked down in tanycytes cells, we sought to investigate whether it caused any alteration in the amount of food intake. At the top of the Fig. 6 the experimental procedure is showing. As shown in Fig. 6A, GK knockdown animals showed a significant increase in food intake over 12 h compared to both control groups. Specifically, GK knockdown animals ate 16 g of food pellet, whereas AdshβGal-EGFP-injected animals ate 11 g; unoperated animals ate 10 g. No significant differences were detected when comparing both control groups (unoperated and Ad-shβGal-EGFP).

To further address a role of GK in feeding behavior, the time that three group of animals spent eating (after fasting) was measured using a system that allowed individual register ad libitum feeding and daily recording of food intake. During the first meal, unoperated animals spent 22 ± 8 min eating, and Ad-shβGal-EGFP-injected animals spent 35 ± 5 min eating. Ad-shGK-EGFP-injected animals spent 78 ± 22 min eating, which was highly significant compared with both control groups, no differences were observed between control groups, suggesting a recuperation of the feeding behavior (Fig. 6B). The meal frequency in GK knockdown animals (expressed as a number of meal events in 12 h) was approximately double the frequency found in the control groups (Fig. 6C). In addition, the cumulative eating time was also increased in Ad-shGK-EGFP-injected animals (Fig. 6D). Although the number of intervals was not affected (Fig. 6E), the interval duration per animal was decreased in GK knockdown animals, indicating a decreased satiety in this group (Fig. 6F). No differences were detected between non-injected and Ad-shβGal-EGFP-injected animals in all parameters.

## Discussion

The hypothalamus is involved in the control of feeding behavior due to its ability to detect and respond to changes in blood glucose levels^30^. Particularly the AN, which is located in the basal region of the hypothalamus, is proximal to the ME, a circumventricular organ known to play an important role in homeostatic regulation^31^. Interestingly, tanycytes link the ventricular and the vascular compartments of the ME and regulate the permeability properties of the fenestrated capillaries of the ME^31^. Unlike the fenestrated portal vessels of the ME, which are permeable to molecules coming from the bloodstream, adjacent tanycytes present various types of tight junctions, which prevent diffusion of substances to the rest of the brain through the CSF. In this manner, blood-borne molecules enter the parenchyma of ME through the permeable vessels, and β2-tanycytes control their diffusion to the CSF via their tight junctions^31^. A different subpopulation of tanycytes, β1-tanycytes, is in close contact with hypothalamic neurons, particularly with the glucose-responsive populations of the AN. This particular distribution of hypothalamic tanycytes represents a key point in the regulation of food intake, and we have previously demonstrated that tanycytes express GLUT2 in their proximal pole (in contact with CSF) as well as GK and ATP-sensitive potassium (K_ATP_) channels^12^.

GK, which catalyzes the high-K_m_ phosphorylation of glucose, is a crucial enzyme in glucose homeostasis in different tissues involved in glucosensing. It is important to highlight that hexokinase I is also expressed in glucose-sensing cells from the VMH. However, its higher affinity for glucose (Km of 1 mM) and kinetic properties prevent hexokinase I from modulating its activity according to changes in glucose levels. Therefore, hexokinase I from the VMH seems to be involved in the continuous generation of ATP regardless of changes in extracellular glucose concentrations whereas GK acts as a glucose sensor^32, 33^. Studies in GK heterozygous knockout mice have attributed a major role for GK in food intake as well as in reproductive functions^34^. In a manner similar to that reported for pancreatic β-cells, GK is required for active glycolysis in β1-tanycytes. An increase in glucose concentration generates an enhanced glycolytic flux, which leads to ATP release in cultured tanycytes^3^. ATP activates P2Y receptors, which provokes an increase in cytosolic calcium levels at the expense of intracellular calcium sources^3^. Interestingly, ATP release and intracellular calcium increase were inhibited in experiments carried out with alloxan, a GK pharmacological inhibitor, demonstrating the importance of GK in this glucose-driven signaling mechanism^3^.

Based on this mounting evidence, the aim of the present study was to knock down GK expression in tanycytes and evaluate the effects of GK suppression on feeding behavior. For this purpose, we generated a GK shRNA-containing adenovirus. Building on the success of previous studies from our laboratory demonstrating the inhibition of hypothalamic MCT1 mRNA expression using adenovirus-mediated transduction of a shRNA into the 3V and its effects on food intake, we focused on the use of this experimental tool in the present study^29^. We demonstrated that the adenoviral vectors selectively and effectively transduced tanycytes from the basal wall of the 3V, which is similar to Xu *et al*.^35^ and Elizondo *et al*.^29^. Additionally, we confirmed that GK mRNA expression was inhibited by 75% with respect to the control. Interestingly, we observed that GK knockdown increased food intake and altered feeding behavior, which is in accordance with a previous study showing that 4V injection of alloxan provokes an increase in food intake^36^. Alloxan is a β-cytotoxic toxic glucose analogue and is commonly used for the development of animal model of Type-I Diabetes Mellitus^37^. Alloxan is rapidly taken up by the pancreatic β-cells through GLUT2^38^. Subsequent studies showed alloxan injected into 3V but not 4V produced a decreased food intake and an altered glucoprivic response, which was accompanied by a massive loss of tanycytes and a decrease in orexigenic NPY^23^, which is related to the presence of GLUT2 in the tanycytes^12^. We could not assess whether the adenovirus reaches more distant brain regions. Further studies are required to analyze whether adenovirus-mediated GK suppression affects the dorsal vagal complex, another cerebral region with important functions as central glucosensing functions^39, 40^. Importantly, it has recently been described that neurons in the nucleus of the solitary tract respond to acutely altered glucose concentration with either increases or decreases in neural excitability and altered synaptic input^24, 39^, which are GK-dependent^40^.

The levels of the anorexigenic peptides, POMC and CART, are known to increase in hyperglycemic conditions, whereas the levels of the orexigenic peptides, NPY and AgRP, decrease in the same conditions. Our results confirmed this pattern of neuropeptide expression in glucose-challenged control rats. However, no differences in the expression of AgRP, NPY and CART were observed between the saline-treated and glucose-treated GK knockdown animals. Interestingly, POMC expression actually decreased in glucose-treated GK knockdown animals. These results are congruent to (and may be the cause of) the augmented food intake as well as the decreased satiety observed in the GK knockdown animals in our study. However, GK inhibition in the AN has recently been shown to decrease food intake in rats^25^. These findings may indicate that the role of GK on food intake is cell type-dependent in a manner similar to that observed for GKRP, which is present in tanycytes, and exerts opposing effects on GK compartmentalization compared to that observed in hepatocytes^15^.

Abnormal feeding initiation and termination after fasting periods due to a failure in the glucosensing mechanism has been reported for GLUT2-knockout mice subjected to fasting/feeding cycles^28^. GLUT2-dependent sensors regulate glucagon secretion under glucoprivic conditions^41^. Thus, GLUT2 and GK expression in tanycytes may have important physiological consequences. A high Km for glucose transport and the presence of GK suggest that tanycytes may increase glucose uptake and metabolization rates proportionally to changes in blood glucose levels^18^. In this regard, GLUT2 is the only glucose transporter with a high Km that has been probed identified to have a role in glucosensing. Among the four known hexokinase isoforms, GK is the only one with a Km in the order of millimolar, which is necessary for participating in the glucosensing mechanism. It is interesting to note the possibility that different populations of tanycytes could detect glucose differently. β2 tanycytes contact not only de CSF but also the fenestrated capillaries (without barrier) of the median eminence, whereas β1 and α tanycytes processes contact blood vessels with barrier. In this regard, it has been demonstrated that β2 tanycytes are able to uptake hormones such a s ghrelin and leptin from the capillaries of the ME and then transfer them to β1 and α tanycytes, which release the hormones in close proximity to their target, the AN neurons^42–44^. Based on this evidence, it is feasible to propose that β2 tanycytes concentrate glucose in the CSF of the infundibular recess in order to be transferred to β1 and α tanycytes, being these populations the responsible of communicating glucose concentration to NA neurons. Of note, this glucosensing mechanism requires a rapid increase in CSF glucose levels, making it possible for the GK-positive glucosensing cells to generate acute responses.

**Figure 7:**
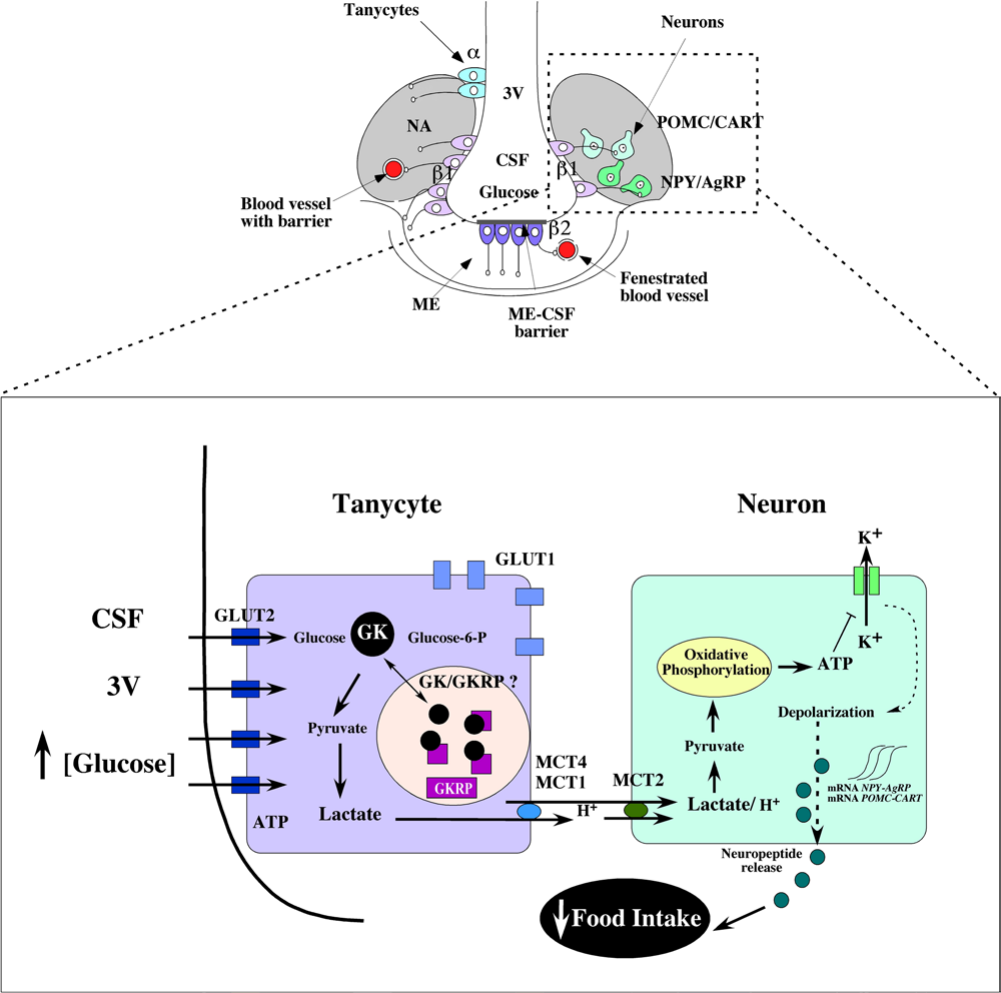
Hypothalamic glucosensing and its inhibition by GK knockdown in tanycytes. Model of glucosensing based on the metabolic interaction between tanycytes and AN neurons. Tanycytes are a specialized ependymal cell, localized in the lower parts of the ventricular walls and the floor of the 3V. α and β1-tanycytes line the infundibular recess, and their basal projections reach orexigenic and anorexigenic AN neurons. β2-tanycytes cover the floor of the 3V and extend their projections inside the ME and regulate the permeability properties of the fenestrated capillaries of the ME. Hyperglycemia results in increased glucose concentrations in the 3V, which affects GK-positive β1-tanycytes. These glial cells sense the increased glucose by the presence of GLUT2 and GK. The GK activity could be regulated by GKRP and its nuclear compartmentalization. Lactate released by tanycytes could activate neurons of AN, generating a satiety response. GK inhibition represses the glucosensing circuit, causing an altered feeding behavior. 3V, third ventricle; ME, median eminence; CSF, cerebrospinal fluid; GK, glucokinase; AN, arcuate nucleus; MCT, monocarboxylate transporter.

For this reason, inhibition of GK expression in β1-tanycytes suppressed the glucosensing network, altering satiation and/or satiety signal processing by neurons from the AN. These results, together with those shown by Elizondo *et al*.^29^ in MCT1 knockdown animals, represent a clear evidence of the metabolic coupling existing between tanycytes and neurons. In this metabolic interaction, tanycytes would metabolize glucose to lactate, and the lactate released by tanycytes could be sensed by neurons as a measure of blood glucose concentration (Fig. 7). It is possible that the astrocyte-neuron lactate shuttle could work as a compensating mechanism for GK inhibition. However, our results indicate that it fails to completely compensate for the effect of GK inhibition.

In conclusion, this is the first evidence of a crucial role for tanycytic GK in feeding behavior and represents an important step in unraveling the hypothalamic glucosensing mechanism^36^ and the way this glucose sensor modulates food intake. These results open a new window in the search for therapeutic strategies for controlling feeding behavior and the treatment of obesity, including the function of the glial element.

## Materials and Methods

### Ethics statement

All animals were handled in strict accordance with the Animal Welfare Assurance (permit number 2010101 A), and all animal work was approved by the appropriate Ethics and Animal Care and Use Committee of the Universidad de Concepción, Chile. Fifty-six male adult Sprague-Dawley rats of 250–300 g were used in all experiments. Animals were housed in a separate animal room with constant temperature (21 ± 2 °C) and a controlled 12-h light/12-h dark cycle; lights were turned on every day at 7:00 a.m. Animals had free access to a standard rodent diet (Lab Diet, 5P00 Prolab RMH 3000, Purina Mills, St. Louis, MO) and tap water.

### Preparations of adenovirus expressing shRNA specific for GK (Ad-shGK)

Serotype 5 ΔE1, E3 based replication-deficient adenoviruses were generated as previously described^29^. Briefly, oligonucleotides targeting rat GK were designed and selected using the Genebank accession number, L38990. Sense shRNA-GK 5′-TCA GAG TGA TGC TGG TCA A-3′ and antisense shRNA-GK 5′-TTG ACC AGC ATC ACT CTG A-3′ sequences shared no homology with other rat coding sequences by BLAST analysis. A ring sequence of nine base pairs (TTC AAG AGA) existed between the sense and antisense strands. Control shRNA oligonucleotides were designed and selected to target β-galactosidase from *E*. *coli*: sense5′-CGC GCC AAG GCC AGA CGC GAA TTA TTT CAA GAG AAT AAT TCG CGT CTG GCC TTT TTT TTT TAA T-3′ and antisense 5′-TAA AAA AAA AAG GCC AGA CGC GAA TTA TTC TCT TGA AAT AAT TCG CGT CTG GCC TTG G-3′. The expression cassette was then cloned into the adenoviral shuttle vector. All shRNAs (Invitrogen, Rockville, MD, USA) were synthesized and designed to contain both AscI and PacI restriction enzyme sites (New England Biolabs, Ipswich, MA, USA), which were used to ligate them into the plasmid, pDC311.2-OFF-EGFP downstream of the human H1 promoter, as previously described^29^. The plasmid was then cotransfected with the Ad genomic plasmid, pBHGlox∆E1,3Cre (Admax system, Microbix Biosystems, Ontario, Canada) into HEK293A cells. Virus particles were released by heat shock, and cell debris was removed by centrifugation for 5 min at 5000× g. The particles were recovered from the supernatant by filtration through a 0.45-µm filter. The resulting adenoviral expression vectors (Ad-shGK-EGFP and Ad-shβGal-EGFP) were tittered by EGFP expression using the Adeno-XTM Rapid Titer Kit Protocol (Clontech, Mountain View, CA, USA).

### Cannula implantation

Rats were handled everyday for one week prior to the experiments to get used to the researchers and the experimental procedures. Rats were anesthetized with an intraperitoneal injection of ketamine (90 mg/kg) and xylazine (10 mg/kg). The fur at the top of the head was removed to expose the area to be incised. A hole was drilled in the skull, and a guide cannula (28 gauge stainless steel; Plastics One, Roanoke, VA) was stereotactically implanted in the 3V of the rat (anterior-posterior from bregma −3.14 mm, medial-lateral from midsaggital sinus 0.0, and dorsal-ventral from the top of the skull 9.2 mm). The guide cannula was secured to the skull using 3/32 mm mounting screws and dental acrylic. A removable dummy cannula (28 gauge stainless steel; Plastics One,) fit into the cannula guide and sealed the opening of the guide cannula throughout the experiments except when it was removed for the injections. After the surgery, rats were single-housed and allowed to recover for 5 days before adenovirus injection.

### Intracerebroventricular (i.c.v.) injections of adenoviruses and immunohistochemistry

Rats were anesthetized with isoflurane and then injected into the 3V with either 25 µL of the control Ad-shβGal-EGFP stock solution (10^8^ IFU/mL) or 25 µL of the Ad-shGK-EGFP stock solution (2 × 10^8^ IFU/mL) for the experimental group at 2.5 µL/min. We were not able to further concentrate the adenovirus in order to inject it in a lower volume. Brains were collected for immunohistochemistry at 18, 24 and 48 h. The rat brains were fixed in 4% paraformaldehyde (PFA) by immersion for 48 h. After fixation, thick frontal sections of the hypothalamus (40 µm) were cut with a cryostat and subsequently processed free-floating. Tissues were immunostained with mouse anti-vimentin (1:200, DAKO, Carpinteria, CA, USA), rabbit anti-glial fibrillary acidic protein (GFAP; 1:200, DAKO) and rabbit anti-NeuN (1:1000, Abcam, Cambridge, MA, USA) antibodies diluted in Tris-HCl buffer (pH 7.8) containing 8.4 mM sodium phosphate, 3.5 mM potassium phosphate, 120 mM sodium chloride, and 1% bovine serum albumin. Sections were incubated with the primary antibodies overnight at room temperature in a humid chamber. After extensive washing, sections were incubated for 2 h at room temperature with Cy2-,Cy3- or Cy5-labeled secondary antibodies (1:200; Jackson ImmunoResearch, West Grove, PA, USA). These samples were counterstained with the DNA stain TOPRO-3 (1:1000; Invitrogen). The slides were analyzed using confocal laser microscopy (LSM 700, Zeiss). Colocalization of different markers was assessed by measuring the Pearson’s correlation coefficient, Rr. An Rr value of ‘1’ indicates complete colocalization, and a Rr value of ‘0’ indicates no specific colocalization.

### Quantitative Reverse Transcription-Polymerase Chain Reaction (qRT-PCR)

The tissues were processed for qRT-PCR analysis as previously described^28, 29^. Briefly, 48 h after adenovirus injection, rats were fasted for 24 h. After that, rats were anesthetized with isoflurane and i.c.v. injected with 10 μL of saline buffer (128 mM NaCl, 3 mM KCl, 1.3 mM CaCl_2_, 1.0 mM MgCl_2_, 1.3 mM NaH_2_PO_4_, 21 mM Na_2_HPO_4_, pH 7.4 and 320 mOsm) or 10 μL of 50 mM D-glucose diluted in the same buffer (320 mOsm, pH 7.4) at a rate of 2.5 μL/min. Hypothalamic samples were collected 2 h post-glucose or saline injection for the mRNA analysis expression. qRT-PCR analysis was used to measure the expression of hypothalamic cyclophilin (the housekeeping gene), GK, NPY, CART, POMC and AgRP. After the experimental treatments, the brain of each rat (six per condition) was removed, and the hypothalamic area was isolated and further dissected to obtain a region close to the 3V ependymal layer. All microdissections were performed closest to the basal 3V and under a stereomicroscope Leica M80 (Leica, Wetzlar, Germany). Total RNA from hypothalamus was isolated using TRIzol (Invitrogen) and treated with DNase I (Fermentas International INC, Burlington, Ontario, Canada) to remove genomic DNA contamination. A total of 2 µg of RNA from each sample was reverse transcribed into cDNA according to the manufacturer’s protocol of M-MULV reverse transcriptase (Fermentas International INC). Parallel reactions were performed in the absence of reverse transcriptase to control for the presence of genomic DNA. qRT-PCR reactions were prepared with a Brilliant II SYBR Green QPCR Master Mix kit (Agilent Technologies, Inc., Santa Clara, CA, USA) in a final volume of 12.5 µL containing 1x SYBR green Master Mix, 1 µL cDNA sample and 500 nM of the following sets of primers: cyclophilin, sense 5′-ATA ATG GCA CTG GTG GCA AGT C-3′ and antisense 5′ATT CCT GGA CCC AAA ACG CTC C3′ (expected product of 239 bp); GK, sense 5′AAA GAT GTT GCC CAC CTA CGT GCG3′ and antisense 5′ATC ATG CCG ACC TCA CAT TGG C3′ (expected product of 510 bp); NPY, sense 5′-TGT TTG GGC ATT CTG GCT GAG G-3′ and antisense 5′- CTG GGG GCA TTT TCT GTG CTT TC-3′ (expected product of 203 bp); AgRP, sense 5′GCA GAC CGA GCA GAA GAT GTT C3′and antisense 5′GTA GCA CGT CTT GAA GAA GC GG3′ (expected product of 186 bp); POMC, sense 5′CTC CTG CTT CAG ACC TCC ATA GAC3′ and antisense 5′AAG GGC TGT TCA TCT CCG TTG3′ (expected product of 164 bp) and CART, sense 5′TCT GGG AAG AAG AGG GAC TTT CGC3′and antisense 5′TCC ATT TGT GTT GCT TTG GGG TG3′ (expected product of 137 bp). All reactions were performed with an initial denaturation of 5 min at 95 °C, followed by 40 cycles of 30 s at 95 °C, annealing for 30 s at 55 °C, and extension for 1 min at 72 °C in an Mx3000 P qPCR System (Agilent Technologies). Ct values of GK mRNA obtained from three different experiments were normalized according to the 2^−ΔΔCt^ method, using cyclophilin as a reference gene^45^.

### Immunoblotting

For protein analysis, hypothalamic samples were collected 48 h post-adenoviral injection. Rat hypothalamus was homogenized in buffer A (0.3 mM sucrose, 3 mM DTT, 1 mM EDTA, 100 μg/mL PMSF, 2 μg/mL pepstatin A, 2 μg/mL leupeptin and 2 μg/mL aprotinin) and sonicated three times on ice at 300 W (Sonics & Material INC, VCF1, Connecticut, USA) for 10 s. After centrifugation at 4,000× g for 10 min, the proteins were resolved by SDS-PAGE (50 µg/lane) in a 12% (w/v) polyacrylamide gel, transferred to PVDF membranes (0.45 µm pore, Amersham Pharmacia Biotech., Piscataway, NJ, USA), and probed with rabbit anti-GK (1:100, Santa Cruz Biotechnology, CA, USA), rabbit anti-GKRP (Santa Cruz Biotechnology), rabbit anti-GLUT2 (Alpha Diagnostics, 1:1000), rabbit anti-EGFP and anti-β-actin (1:1000) antibodies. After extensive washing, the PVDF membranes were incubated for 1 h at 4 °C with peroxidase-labeled anti-rabbit IgG (1:5000; Jackson ImmunoResearch). The reaction was developed using an enhanced chemiluminescence (ECL) Western blotting analysis system (Amersham Biosciences). Negative controls consisted of incubating the membrane in the absence of anti-GK. The images shown are representative of at least three analyses performed on samples from at least three separate experiments. β-actin expression levels were used as a loading control, and GFP expression levels were used as a transduction control.

### Food intake monitoring and feeding behavior analysis

At 24 h post-adenoviral injection, rats were subjected to a 24 h fasting period followed by a 12 h refeeding period for the feeding behavior analysis. Food intake was monitored during the refeeding period by providing rats with pre-weighed rat chow and weighing it again after 12 h (Fig. 6, schematic representation). Food intake was expressed as g consumed per 200 g of body weight. Every interaction with the feeder was recorded by a computerized data acquisition system (VitalView, Respironics, Inc., Murraysville, PA, USA), registering frequency and time of permanency in the feeder.

A meal was considered when the bouts were larger than 10 s into the feeder, and these meals were separated from other feeding bouts by more than 10 min of inter-meal interval^46, 47^. Except for the first meal, when a bout was longer than 30 min, two meals were considered. The meal patterns calculated included the following: duration of the first meal (in min), frequency of meals (in number of meals in 12 h), cumulative eating time as the total time spent in the feeder (in min), duration of inter-meal intervals (in min), and number of meal intervals. The inter-meal interval was calculated as the time period between the end of one meal and the initiation of the next one. The mean meal size was determined as the total food intake (g) divided by frequency. The mean meal duration was calculated by dividing the total meal duration (in min) by meal frequency, and the eating rate was estimated dividing total food intake (mg) by meal duration (min).

### Statistical analyses

For each data group, results were expressed as mean ± standard error of the mean (SEM), and *n* refers to the number of animals that were used. For statistical analysis, each treatment was compared with its respective control. Differences between two groups were assessed using the Student t-test. Differences between more than two groups were assessed using ANOVA. Differences were considered significant when *P* < 0.05. The statistical analyses were performed using GraphPad Prism 5.0 Software (GraphPad Software Inc., San Diego, CA, USA).

## Electronic supplementary material

Figures Supplementary
