# Author Correction: Adenovirus-mediated suppression of hypothalamic glucokinase affects feeding behavior

**DOI:** 10.1038/s41598-019-48627-x

**Published:** 2019-08-28

**Authors:** Romina María Uranga, Carola Millán, María José Barahona, Antonia Recabal, Magdiel Salgado, Fernando Martinez, Patricio Ordenes, Roberto Elizondo-Vega, Fernando Sepúlveda, Elena Uribe, María de los Ángeles García-Robles

**Affiliations:** 1 Departamento de Biología Celular, Universidad de Concepción, Concepción, Chile; 2 Departamento de Bioquímica y Biología Molecular, Facultad de Ciencias Biológicas, Universidad de Concepción, Concepción, Chile; 3 Instituto de Investigaciones Bioquímicas de Bahía Blanca, Universidad Nacional del Sur, y Consejo Nacional de Investigaciones Científicas y Técnicas, Bahía Blanca, Argentina; 4 Facultad de Artes Liberales, Facultad de Ingeniería y Ciencias, Universidad Adolfo Ibáñez, Viña del Mar Chile, Chile

Correction to: *Scientific Reports* 10.1038/s41598-017-03928-x, published online 16 June 2017

The Acknowledgements section in this Article is incomplete.

“The authors thank Dr. Marjet Heitzer for helpful discussion and suggestions on the manuscript.”

should read:

“Grant sponsor: FONDECYT; Grant number: 1140677(MAGR), 11150992(FS) and 11121518(CM). The authors thank Dr. Marjet Heitzer for helpful discussion and suggestions on the manuscript.”

