# Supplementary material for: Adenovirus-mediated suppression of hypothalamic glucokinase affects feeding behavior
Source: Sci Rep. 2017 Jun 16;7:3697. doi: 10.1038/s41598-017-03928-x (PMC5473813; doi:10.1038/s41598-017-03928-x)
Supplement: Supplementary file 1 — Figures Supplementary [file 41598_2017_3928_MOESM1_ESM.pdf]

Adenovirus-mediated suppression of hypothalamic glucokinase affects feeding behavior

Romina María Uranga[1,3]*, Carola Millán[1,4]*, María José Barahona[1], Antonia Recabal[1], Magdiel Salgado[1], Patricio Ordenes[1], Roberto Elizondo-Vega[1], Fernando Sepúlveda[1], Elena Uribe[2] and María de los Ángeles García-Robles[1&]

1 Departamento de Biología Celular, 2 Departamento de Bioquímica y Biología Molecular, Facultad de Ciencias Biológicas, Universidad de Concepción

3Instituto de Investigaciones Bioquímicas de Bahía Blanca, Universidad Nacional del Sur, y Consejo Nacional de Investigaciones Científicas y Técnicas, Bahía Blanca, Argentina

4Facultad de Artes Liberales, Facultad de Ingeniería y Ciencias, Universidad Adolfo Ibáñez, Viña del Mar Chile

& Address correspondence and reprint requests to M A. García-Robles, Departamento de Biología Celular, Facultad de Ciencias Biológicas, Universidad de Concepción, Casilla 160-C, Concepción, Chile

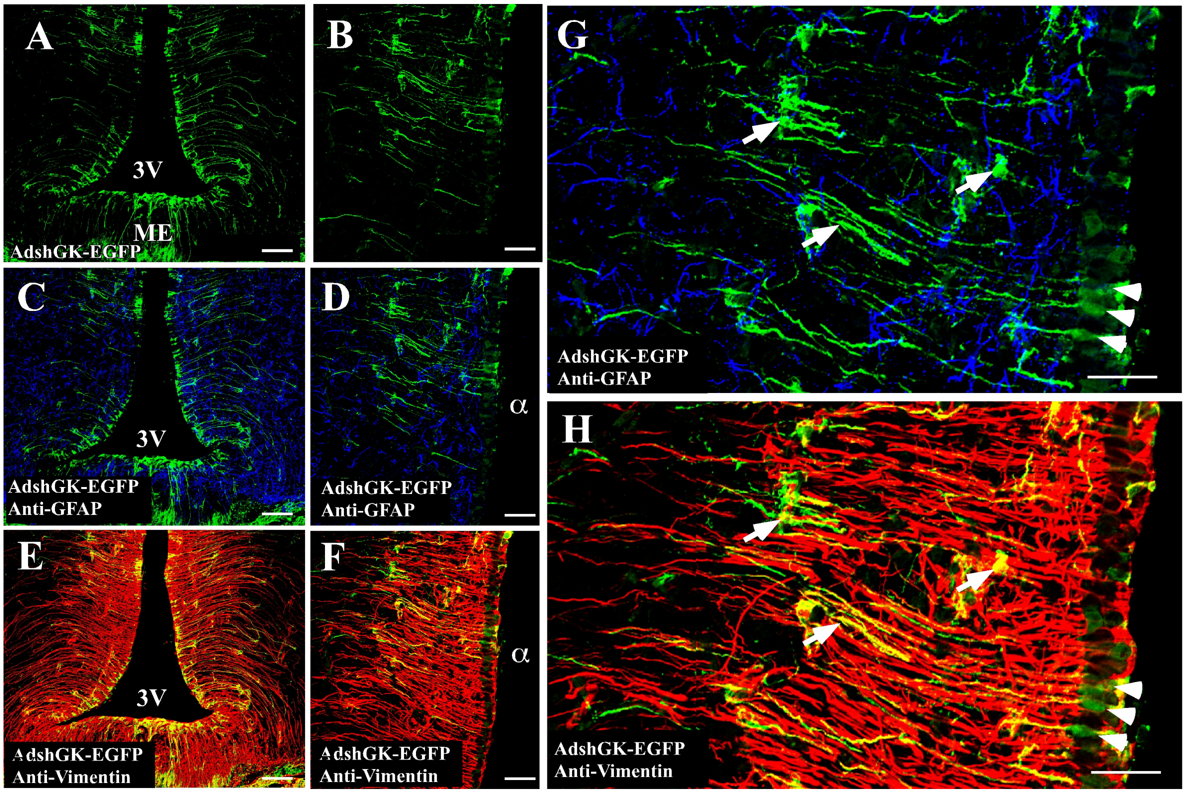

Supplementary Figure 1. Hypothalamic GFAP expression after Ad-shGK-EGFP injection into the 3V. Confocal microscopy analysis of vimentin (red), GFAP (blue) and EGFP (green) associated fluorescence **A-B,** EGFP is detected in apical membranes and long cellular processes in the basal hypothalamus. **C-D,** showing astrocyte distribution and the absence of EGFP colocalization. **E-F,** Vimentin showing tanycyte distribution and colocalization with the adenovirus. **G-H,** High magnification images showing astrocytes negative for EGFP (G) and tanycytes positive for EGFP (H). Scale bar A-F: 50 μm; G-H: 25 μm.

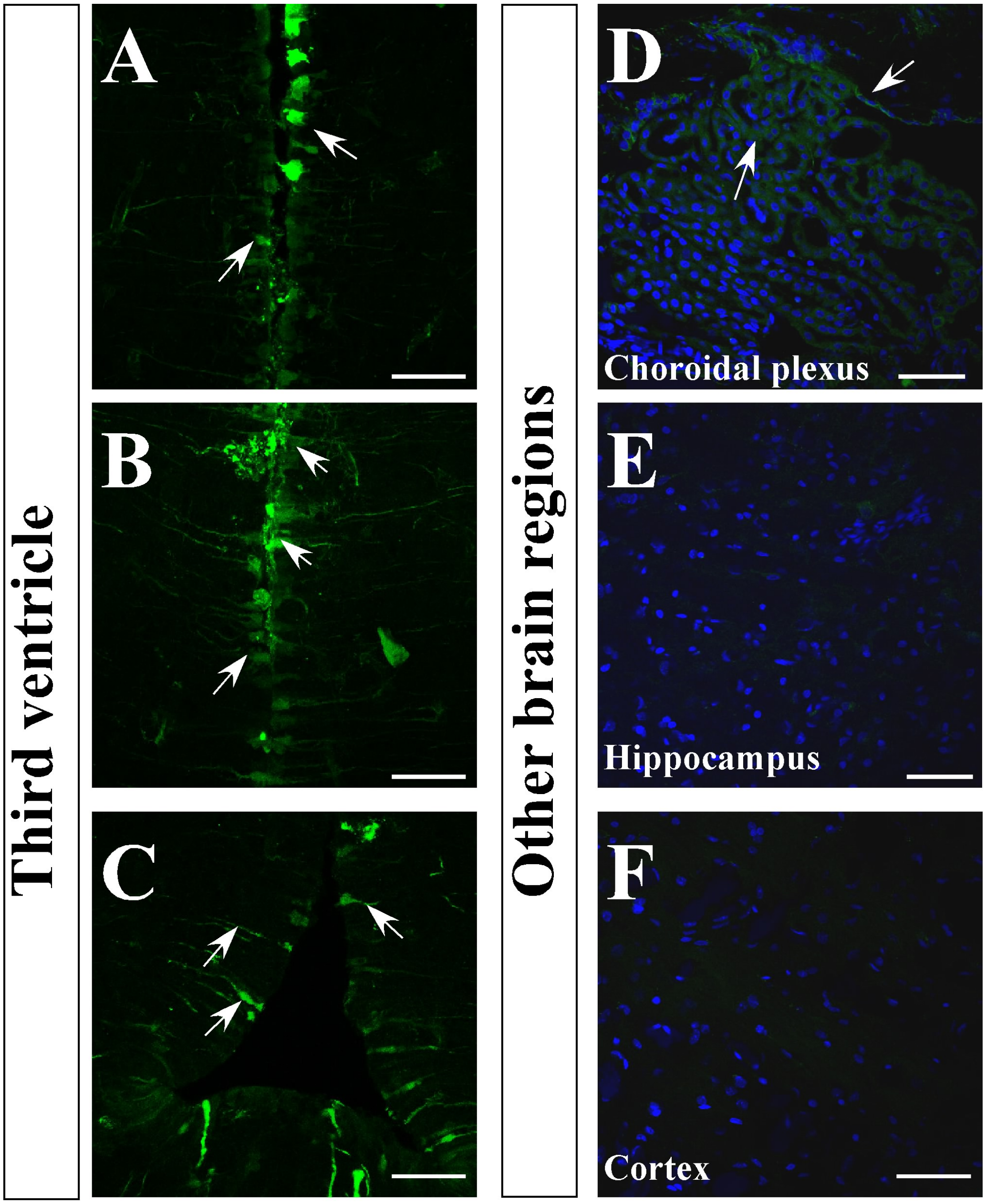

Supplementary Figure 2. Transduction of Ad-shGK-EGFP in the third ventricle and other brain regions. **A-C,** EGFP expression in the dorsal (A), medium (B), and basal regions of the 3V (C). **D-F**, EGFP expression in chroroidal plexus (D) showing low intensity, hippocampus (E) and cerebral cortex (F); EGFP was not detected in the last two areas. Scale bars: 50 µm

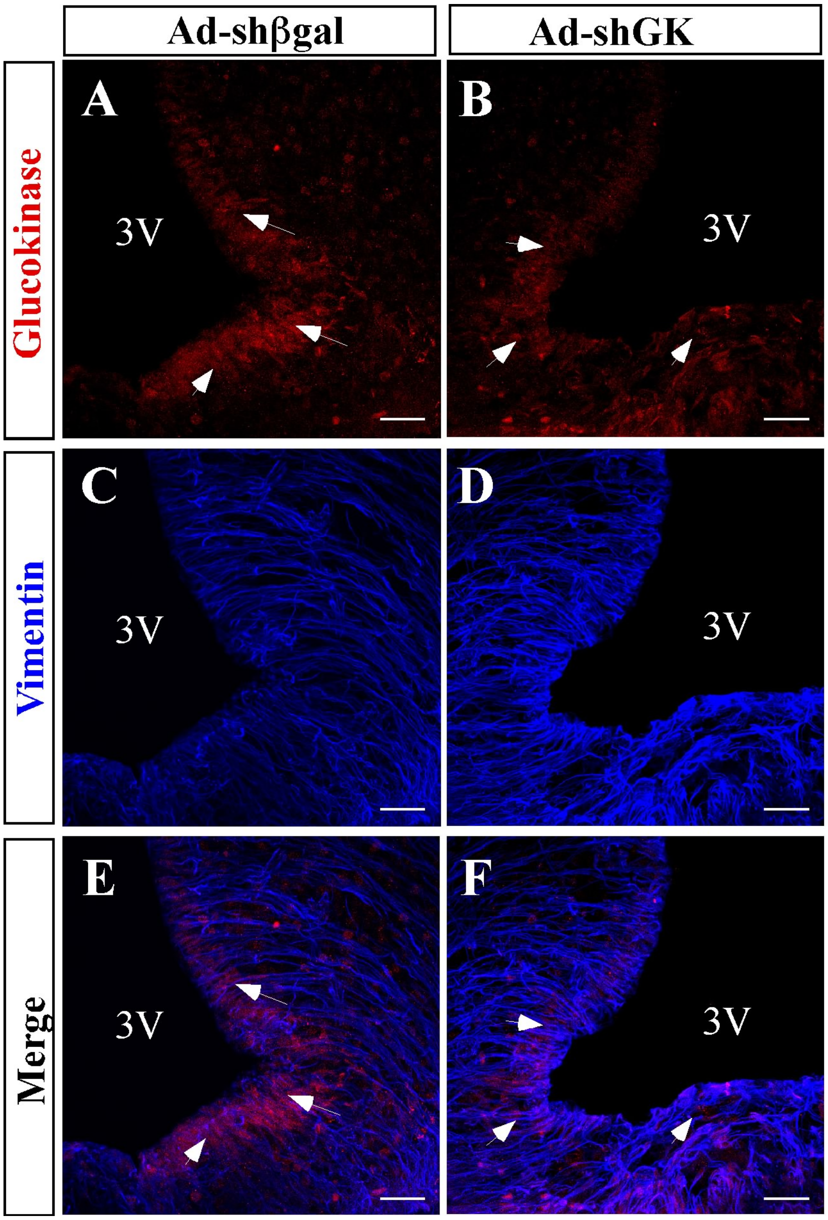

Supplementary Figure 3. GK expression at 48 h after Ad-shGK-EGFP injection into the third ventricle. Frontal sections from the hypothalamus at 48 h after i.c.v. injection of Ad-shβGal (A,C and E) and Ad-shGK (B,D and F). Immunoreactivity for GK is in red and nuclear marker, TOPRO 3, is in blue. Scale bars: 50 μm.
